# Supplementary material for: Splice-Junction-Based Mapping of Alternative Isoforms in the Human Proteome
Source: Cell Rep. Author manuscript; Available in PMC 2020 Jan 15. (PMC6961840; doi:10.1016/j.celrep.2019.11.026)
Supplement: 2 [file NIHMS1546469-supplement-2.zip › DF1/PXD006675/SmoothMuscleCells/SmoothMuscleCells_10_MAP4_KPCSETSQIEGSPTEFLEEK.pdf]

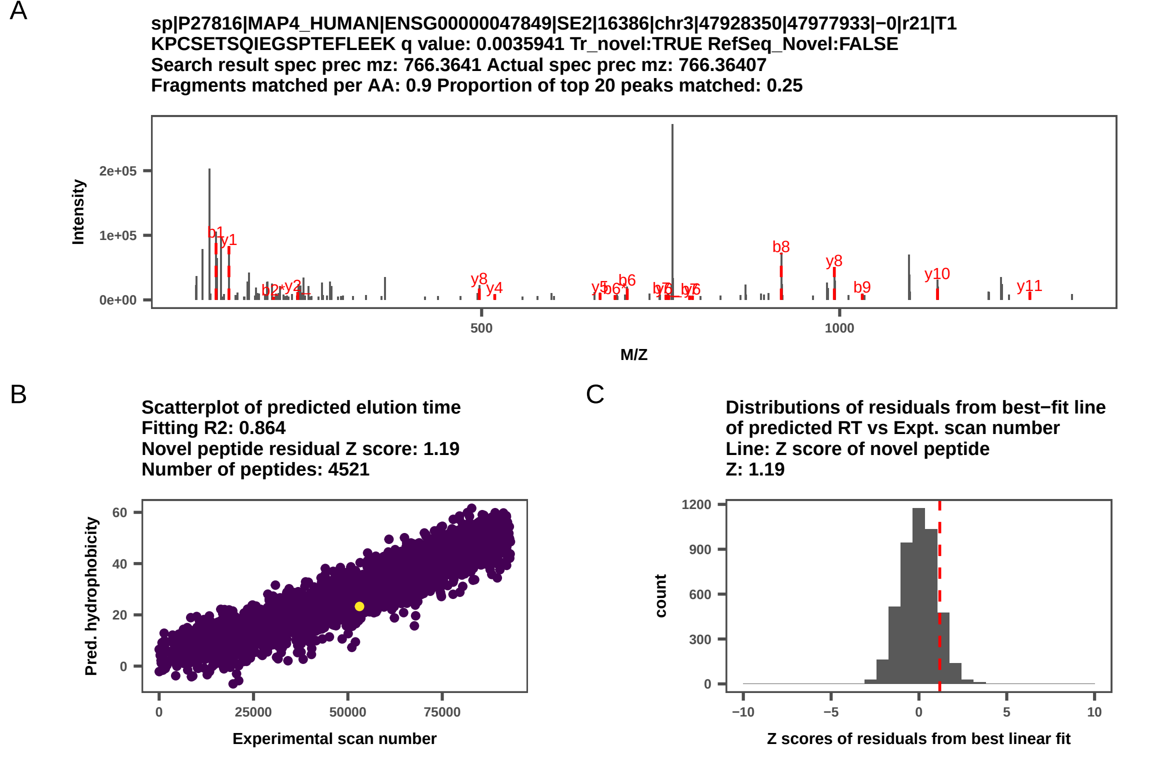

A
sp|P27816|MAP4_HUMAN|ENSG00000047849|SE2|16386|chr3|47928350|47977933|−0|r21|T1
KPCSETSQIEGSPTEFLEEK q value: 0.0035941 Tr_novel:TRUE RefSeq_Novel:FALSE
Search result spec prec mz: 766.3641 Actual spec prec mz: 766.36407
Fragments matched per AA: 0.9 Proportion of top 20 peaks matched: 0.25
Intensity
2e+05
1e+05
0e+00
b1
y1
y8
y4
b6
b8
y8
b9
y10
y11
500
1000
M/Z
B
Scatterplot of predicted elution time
Fitting R2: 0.864
Novel peptide residual Z score: 1.19
Number of peptides: 4521
Pred. hydrophobicity
60
40
20
0
0
25000
50000
75000
Experimental scan number
C
Distributions of residuals from best−fit line
of predicted RT vs Expt. scan number
Line: Z score of novel peptide
Z: 1.19
count
1200
900
600
300
0
−10
−5
0
5
10
Z scores of residuals from best linear fit
